# Household exposure to violence and human rights violations in western Bangladesh (I): prevalence, risk factors and consequences

**DOI:** 10.1186/1472-698X-9-29

**Published:** 2009-11-21

**Authors:** Shr-Jie Wang, Jens Modvig, Edith Montgomery

**Affiliations:** 1 Rehabilitation and Research Centre for Torture Victims (RCT), Copenhagen, Denmark

## Abstract

**Background:**

The ruling parties in Bangladesh have systematically used violence against political opponents and criminals. It is essential to 1) determine the magnitude and burden of organised crime and political violence (OPV) and human rights violations in the affected community, and to 2) identify the risk factors and key indicators for developing effective health intervention and prevention measures.

**Methods:**

The population-based study consisted of two parts: a household survey and OPV screening at mobile clinics (presented in Part II). A cross-sectional, multistage cluster household survey was conducted in the Meherpur district in February-March 2008; 22 clusters with a sample size of 1,101 households (population of 4,870) were selected.

**Results:**

Around 83% of households reported being exposed to at least two categories of OPV or human rights violations: 29% reported that the family members had been arrested or detained; 31% reported torture or other cruel, inhuman or degrading treatment or punishment. Crude mortality rate was 17.9/1,000 and under 5 mortality rate was 75/1,000. The annual injury rate was 36%, lifetime experience of violence-related injury was 50%, and pain experience within 2 weeks was reported by 57%. Over 80% of the population over 35 years old complained of pain. High prevalence of injury, lifetime experience of OPV-related injury and pain complaints are related to the level of exposure to OPV and human rights violations. A financial burden was imposed on families with an injured person. A geographical variation was revealed regarding reports of torture and lifetime experience of violence-related injury. A combination of individual, relational, community and societal factors, including variables such as political party affiliation, conflict with other families, household income and residential area, affected the risk of victimisation in the household. The odds ratio for reporting extrajudicial execution of a family member was 9.22 for Awami League supporters, 9.15 for Bangladesh Nationalist Party supporters; and 3.97 for Jamaat-e-Islami Party supporters compared with families with no political involvement.

**Conclusion:**

The level of violence and human rights violations is high. The affected population suffers from violence-related injuries and traumas, which could be a factor contributing to poverty. Victimisation is not random.

## Background

Collective violence is categorised by the World Health Organization (WHO) as follows: 1) wars, terrorism and other violent political conflicts; 2) state-perpetrated violence such as genocide, repression, disappearances, torture and other abuses of human rights; 3) organised violent crime such as gang warfare [1]. Organised crime and political violence (OPV) is the most common collective violence in conflict settings. Torture is frequently practiced in the context of OPV and human rights violations. In Bangladesh, no population-based assessment of OPV and human rights violations has yet been conducted with a focus on risk factors and consequences, especially health consequences.

### OPV in Bangladesh

Torture and other serious human rights violations were committed during the Bangladesh Liberation War with Pakistan in 1971, when the authorities specifically targeted political dissidents in Bangladesh [2]. In independent Bangladesh, both the Awami League and the Bangladesh Nationalist Party (BNP) have used armed groups and militias against their political opponents. They have also systematically used torture and ill-treatment as a means to suppress opposition parties and criminals [3-5]. Torture and violent attacks against women have been widespread as well [6-9]. A previous study of refugees from Bangladesh treated at the Centre for Torture and Trauma Survivors in Stockholm reported that all of the female refugees and one-third of the male refugees had been raped [10].

**Figure 1:**
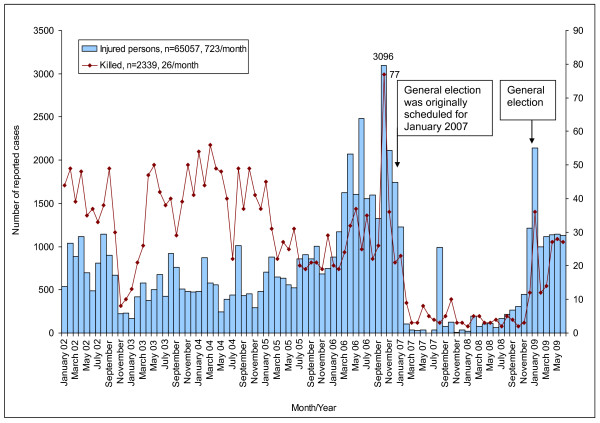
**Reported injury and killings due to political violence in Bangladesh in 2002-June 2009, source: Odhikar **.

The BNP won the election in October 2001 and launched Operation Clean Heart in October 2002 as a drive against growing crime: a rise in murder, rape, and acid throwing. In March 2004, the anti-crime and anti-terrorism elite force 'Rapid Action Battalions' was founded. Up to December 2007, Rapid Action Battalions had seized a total of 23,632 people including two senior terrorists and recovered 3,592 illegal weapons based on official data from their website [11]. Their lack of accountability has been questioned, however, by the Human Rights Watch and Amnesty International [3,4]. Daily violent attacks on the press by political militants, criminal gangs and security services were also reported by Reporters Without Borders [12]. A local non-governmental organisation (NGO), Odhikar, documented human rights abuses by gathering information through a nationwide network of trained human rights workers and from media reports: 65,057 injuries and 2,339 killings due to political clashes were identified during the period January 2002-June 2009 (Figure 1). The number of reported incidents peaked 2 months before the general election in October 2006 at 3,096 injured and 77 killed. The Awami League alleged electoral bias in favour of its rival, the BNP, and boycotted the elections. The president of the caretaker government announced a state of emergency and curfews in January 2007, due to weeks of riots and protests over erroneous voter lists, and postponed the general election until December 2008. Pre- and post-election violence was observed at the end of 2008 and the beginning of 2009.

### Human rights violations in Khulna division

Khulna division, which borders India, is the province most affected by violence in Bangladesh. Operation Spider Web was launched in 2004 by the BNP-led government in the south-western districts of Bangladesh after Operation Clean Heart failed to restore public order. Its primary target was the left-wing extremists along the Indian border. Substantial numbers of extrajudicial killings in Khulna division have been reported since 2004 [3,13,14]. The Bangladesh Rehabilitation Centre for Trauma Victims (BRCT) also reported 1,102 torture cases and 414 torture-related deaths in Khulna division in 2004 [15].

### Rationale for site selection

Meherpur district, with its area of 716.08 square kilometres, is the smallest district in Bangladesh. It is located in Khulna division, and borders West Bengal in India to the north. In 1971, the leaders of the Awami League declared the independence of Bangladesh in the town of Mujibnagar. Throughout the liberation war with Pakistan, the Mujibnagar upazila served as the base for the pro-independence militias. Consequently, the area was severely affected by the war. Meherpur district is currently under the threat of left-wing and Islamic extremism. In the south-western districts of Kushtia, Meherpur, and Chuadanga, where at least six outlawed parties with 4,000 armed members are active, many people were killed and frequent cases of abduction, robbery, rape, murder and other crimes were recorded. In the last 10 years, the BRCT identified and treated 126 cases of torture and organised violence in the Meherpur district. The Task Force against Torture has been working in Meherpur district, and a local NGO, Manab Unnayan Kendra, provides legal and social support to female victims of rape and domestic violence.

### Objectives

This is a multisite epidemiological study whose initial focus is to assess OPV and human rights violations at the population level and to study their causes and consequences at the individual and family level. This study was commissioned by the Rehabilitation and Research Centre for Torture Victims (RCT) for three countries and was implemented in Bangladesh and Kosovo in 2008. A separate review will describe this work in a wider context. The population-based survey was aimed at: 1) describing the magnitude and patterns of OPV and human rights violations; 2) estimating the prevalence of injury, OPV-related injury and pain in the violence-affected area; 3) identifying the risk determinants of victimisation, 4) quantifying the association between OPV exposure and general health conditions, as well as family financial burden. Our hypotheses are as follows: 1) there are spatial variations in levels of OPV and number of reported cases of human rights violations; 2) mortality and prevalence of injury and pain correlate with the level of OPV and human rights violations in the community; 3) a family could be at increased risk of becoming victims of OPV and human rights violations if any of the family members is active in a social or political movement or has trouble in interpersonal relationships.

## Methods

The key components of the research methodology were: 1) collection of statistical data and mapping information; 2) a fact-finding mission and focus group discussion; 3) a population-based study (household survey and subsequent health screening of selected victims of OPV and human rights violations in mobile clinics). A focus group discussion was carried out with the objectives of: building trust within the local community, assessing the local people's perception of OPV victims and collecting public opinion on the causes and consequences of OPV. The findings were used to prepare for in-depth interviews and to design questionnaires for the household survey in the next phase.

### Household survey

A generic protocol was developed based on a WHO document "Guideline for conducting community surveys on injury and violence" [16]. It was modified to focus specifically on OPV-related injury and mortality. Optimal sample sizes were calculated using the standard statistical formula developed by the United Nations Children's Fund (UNICEF) [17]. The formula was as follows: n = [4 (r) (1-r) (f) (1.1)]/[(e^2^) (p) (nh)] with an anticipated prevalence of 15-30% (r), a design effect of 2 (f), a factor necessary to raise the sample size to allow for an estimated non-response rate of 10%: 1.1, a margin of error of 10% (e) of r, the proportion of the total population that the smallest subgroup comprises (p): 1, and an average household size of 4.9 (nh) in Bangladesh. The estimated number of households that needed to be surveyed in order to estimate the prevalence of lifetime experience on violence-related injury in the whole population was around 500-1,000 households.

#### Sample selection and case definition

The 2001 Bangladesh census was used as a sampling frame: in Meherpur district there were 137,901 households with a population of 591,436, which was adjusted using annual population growth rates in the urban area and paurashava (capital) (2.63%) and rural area (1.61%) [18]. In 2008, the total number of households was estimated to be 155,837 with a population of 661,854. Meherpur district consists of three upazilas (Gangni, Meherpur Sadar and Mujibnagar), 18 unions, 190 mauzas, 278 villages, 1 paurashava (capital), 9 paura wards and 72 mahallah. A multistage cluster sampling was used. In the first stage, Meherpur district was stratified into urban and rural areas. The cluster size was 50 households and, therefore, there were 3,117 clusters in Meherpur district. For the household survey, 20 clusters were required with a sampling size of 1,000 households. Two additional clusters were selected for quality control. In the second stage, a random cluster sampling with a probability proportional to size methodology was employed to select individual households. The rural and urban population ratio in Meherpur district is 88:12; thus, 20 rural clusters and two urban clusters were randomly selected. A total of 22 clusters with a total sample size of 1,101 households were visited.

Each interviewer was responsible for two clusters. Households within the village were randomly selected. A starting point in the village was randomly chosen, and the interviewer walked along the street in a random direction. The interviewer visited the households by using an appropriate household sampling interval, which depended on the size of village. The first house was the *n*-th house on the street in the selected direction. If the selected house was empty, the next household was visited. Because of security concerns, sometimes the interviewers avoided the areas near police stations and military camps or crime-affected neighbourhoods. One cluster was completed as 50 households were visited.

A 'household' was defined as a group of people cooking in the same kitchen or sleeping under the same roof. The definition of 'torture and other cruel, inhuman or degrading treatment or punishment' was provided by The United Nations Convention against Torture and Other Cruel, Inhuman or Degrading Treatment or Punishment. The legal definition of 'forced or compulsory labour' was provided by the International Covenant on Civil and Political Rights, Article 8. The Geneva Convention additional protocol II was also referenced; this addressed the protection of objects indispensable to the survival of the civilian population (Article 14) and the prohibition of forced movement of civilians (Article 17). The definition of violence used was adapted from the WHO [1]. Death and injury cases were classified using the WHO [16] and the International Statistical Classification of Diseases and Related Health Problems, 10^th ^edition (ICD-10). Violence-related injury included interpersonal violence, self-directed violence, legal intervention, war, civil insurrection and disturbance (demonstration or riots). Violence-related deaths, therefore, included homicide and suicide. The death within 12 months was reported by the household members. There is no official document for confirmation.

#### Study implementation

A cross-sectional survey was conducted from 23 February 2008 to 10 March 2008. A pre-pilot study in the rural area and a pilot study in Meherpur paurashava (capital) were implemented. The deputy team leaders arrived in the selected village three days before the survey and informed survey participants and the community head about the purpose and procedures in the proposed study. Selected households were then visited and one person (the head or his/her spouse) was recruited and interviewed after providing his/her informed consent. The household members were asked to remain to confirm the information provided. Interviews were carried out using a structured questionnaire covering four themes: 1) social and demographic characteristics; 2) injury and pain suffered by each household member and the overall injury-related financial burden of the household; 3) personal relationships and social participation of household members; 4) household experience with violence and human rights violations. The survey questionnaire was developed in English and translated into Bengali. Each interview took 30-40 minutes; at the end of each day, all answers were reviewed for completeness by the interviewer, deputy team leader and principal investigator. During the household survey, the interviewers were not informed about who would eventually be invited to the mobile clinic. In addition, the survey participants were not informed about the possibility of free examination and treatment in the mobile clinic.

### OPV screening at the mobile clinic

**Figure 2:**
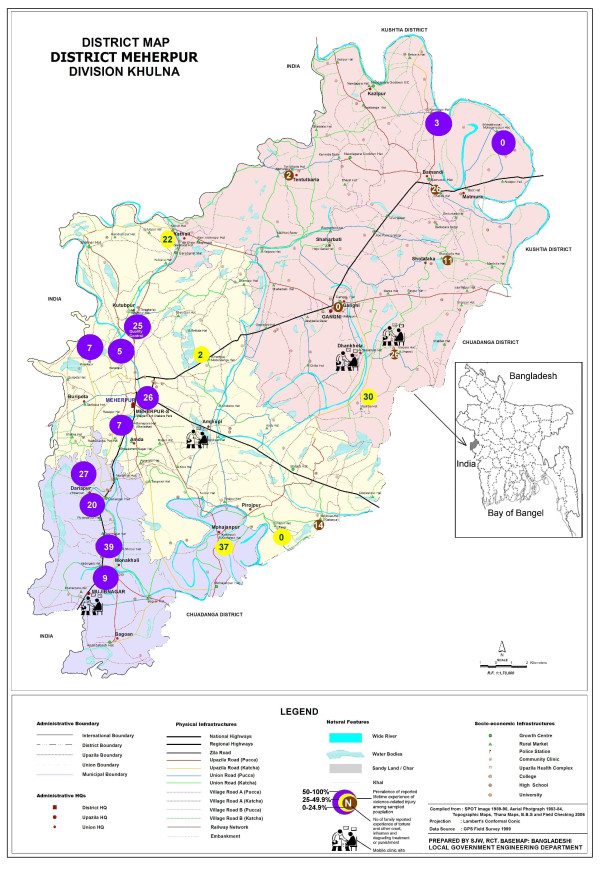
**Spatial variations in the proportion of sampled populations with lifetime experience of violence-related injury and the number of sampled households reporting experience of torture and other cruel, inhuman or degrading treatment or punishment**. February--March 2008, Meherpur district.

When the household survey was finished, the list of families to be invited to the mobile clinic was prepared based on the survey results. The interviewers were told to return to the villages for which they were responsible and distribute the vouchers to the victims' families who reported incidents of: 1) torture and other cruel, inhuman or degrading treatment or punishment; 2) sexual harassment, molestation, rape or insertion of a blunt object into a genital organ and/or rectum; 3) arrest or detention; 4) extrajudicial execution of family member, perpetrated by members of law enforcement agency. The victims were invited to the mobile clinic for free examination and treatment. Two-day mobile clinics were organised at four sites in Meherpur district (Figure 2). The victims were screened and interviewed, and then underwent physical examination and consultation. The results are presented in Part II.

### Quality assurance

Only four households refused or did not complete the interview. During the household survey, every tenth to twentieth of the respondents was interviewed twice by different interviewers or randomly selected for spot-check by deputy team leaders. The data set was checked three times for discrepancies.

### Statistical analysis

Data were entered and validated using Microsoft Access 2000 and Epi Info™ 6.04 (CDC Atlanta, USA, 2001). The data were analysed using Stata 9.2 (StataCorp LP, Texas, USA, 2003) at the individual, household, and village levels. The major explanatory variables were age group, sex, religion, area of residence, education and occupation of head of household, political involvement and social participation of family members, as well as the household income level: 0-1 United States dollars (USD) per day, 1-2 USD per day, 2-4 USD per day, and higher than 4 USD per day. A generalised linear model was used to assess the association between outcomes and explanatory variables, adjusted for the cluster effect (village) and the confounding effects of age groups. P values < 0.05 were considered significant.

### Ethical evaluation

The study complied with the Declaration of Helsinki and Danish law. The Bangladesh NGO Bureau gave approval for the study. In the household survey, the head (or the spouse of the head) of a household provided the informed consent. Confidentiality and privacy protection was respected. Basic treatment was offered to simple cases of torture or ill-treatment identified during the subsequent OPV screening at the mobile clinic. Severe cases were referred to the BRCT. The sponsor had no role in the study design, data collection and analysis, interpretation of the data, or writing of the report.

## Results

### Profile of sampled households and population

The total study population was 4,870: 50 households (4.5%) with 250 persons were located in the capital of Meherpur Sadar upazila (sub-district), 50 households (4.5%) with 217 persons were located in the suburban area in Gangni upazila, and 1,001 households (91.0%) with 4,403 persons were located in rural areas. The average size of a household was 4.4 people (range: 1-15).

The demographic profile of the study population is provided in Tables 1 and 2. The mean age of the study population was 28.0 (range: 0-102), and 8.2% were under 5 years old. Over 96% of heads of households were Muslim and 2.3% were Hindu. More than 50% of heads of households were engaged in agricultural, fishing, animal husbandry or hunting activities; nearly one-fifth were engaged in business, which is much higher than the percentage from the 2001 national census in Meherpur district. The possible explanation is that, in the border area, many people were engaged in trade and smuggling between Bangladesh and India. The total household income ranged from 0 to 36,000 Bangladesh taka per month (0 to 525 USD). Overall, 22.6% of households lived below the poverty line (1 USD per day).

**Table 1:** Social demographic profile of sampled households, n = 1101

Social demographic	Variables	No. of households (%)	Remarks
Upazila (sub-district) of Meherpur district	Gangni upazila	449 (40.8)	341.98 Square km
	Mujibnagar upazila	200 (18.2)	112.68 Square km
	Meherpur Sadar upazila	452 (41.1)	261.42 Square km
	Total	1101 (100)	716.08 Square km

Education level of head of household	None	467 (42.4)	Bangladesh census: 42.1 [18]
	Primary	329 (29.9)	Bangladesh census: 31.7 [18]
	Secondary	209 (19.0)	Bangladesh census: 22.4 [18]
	College or university	51 (4.6)	Bangladesh census: 3.4 [18]
	Post-graduate	9 (0.8)	Bangladesh census: 0.4 [18]
	Other	31 (2.8)	
	Missing	5 (0.5)	

Religion of head of household	Muslim	1058 (96.1)	Meherpur in 2001: 97.6%
	Hindu	25 (2.3)	Meherpur in 2001: 1.1%
	Missing	18 (1.6)	

Occupation of head of household	Not working	24 (2.2)	Meherpur in 2001: 27.5%
	Household work	50 (4.5)	Meherpur in 2001: 33.2%
	Agriculture, fishing, animal husbandry or hunting	603 (54.8)	Meherpur in 2001: 27.4%
	Business	206 (18.7)	Meherpur in 2001: 4.7%
	Service, journalist or teacher	42 (3.8)	Meherpur in 2001: 0.3%
	Other	157 (14.3)	Meherpur in 2001: 6.6%
	Missing	19 (1.7)	

Monthly income of household (Exchange rate: Bangladesh taka: USD = 68:1)	< 2000 taka	249 (22.6)	Poverty line: 1 USD per day
	2000 ≤ × <4000 taka	527 (47.9)	Poverty line: 2 USD per day
	4000 ≤ × < 8000 taka	241(21.9)	2-4 USD per day
	≥ 8000 taka	74 (6.7)	≥ 4USD per day
	Missing	10 (0.7)	

Family member is involved in political party	None	808 (73.4)	
	Awami League	128 (11.6)	
	Bangladesh Nationalist Party (BNP)	127 (11.5)	
	Jamaat-e-Islami Party	31 (2.8)	
	Other	3 (0.3)	
	Missing	4 (0.4)	

Family has personal, financial or political conflict with other families	No conflict	295 (26.8)	
	Yes	803 (72.9)	
	Missing	3 (0.3)	

Family member has ever participated in a demonstration, a strike or a human rights rally	No	606 (55.0)	
	Yes	492 (44.7)	
	Missing	3 (0.3)	

Family has relative or friend working with a law enforcement agency	No	536 (48.7)	
	Yes	562 (51.0)	
	Missing	3 (0.3)	

**Table 2:** Demographic and health indicators of study population, n = 4870

Age group	Male	Female	Total (%)
Under 5	191	209	400 (8.2)
5-14	465	494	959 (19.7)
15-64	1652	1615	3267 (67.1)
≥ 65	136	108	244 (5.0)
Total	2444	2426	4870 (100)

**Mortality**	**No. of people/Total**	**Per 1,000**	**Remarks**

Crude Mortality Rate (03/07-02.08)	87/4870	17.9	UNICEF figure in 2006 (8 per 1,000)
Under 5 Mortality Rate (03/07-02.08)	30/400	75	UNICEF figure in 2006 (69 per 1,000)
Mortality due to violent attack (03/07-02.08)	5/4870	1.02	1.2 per 1000 in eastern Burma in 2002-2003[28]

**Injury and pain experience (self-reported)**	**No. of people/Total**	**% (95% CI)**	**Remarks**

Injury within 12 months	1746/4870	35.9 (34.50-37.20)	
Lifetime experience of violence-related injury	2426/4870	49.8 (48.21-51.22)	
Pain complaints within 2 weeks preceding the survey	2756/4870	56.6 (55.20-57.98)	
Injury due to OPV at some point in lifetime	457/4870	9.4 (8.60-10.23)	
OPV-related injury/overall violence-related injury	457/2426	18.8 (17.30-20.45)	

Table 2 shows the prevalence of injury within 12 months, lifetime experience of violence-related injury, and pain complaints within 2 weeks. The average number of people in each family who reported injury within 12 months was 1.6 (95% CI: 1.51-1.68); for lifetime experience of violence-related injury, the number was 2.2 (95% CI: 2.08-2.32); for pain complaints within 2 weeks, the number was 2.5 (95% CI: 2.4-2.56). Almost half of the population had been injured because of violence at some point in their lifetime and 18.8% of violence-related injury was due to OPV.

In 2007, the crude mortality rate (CMR) in the study population was 17.9 per 1,000 and the under 5 mortality rate (U5MR) was 75 per 1,000 (Table 2). The survey participants reported that at some point in their lifetime, 457 people had been injured and 15 had been killed because of OPV. Of the 87 people who died within 12 months of the survey, five (5.7%) were reported to be killed because of violent attacks; this is 1.02 per 1,000 people. We estimate, therefore, that in 2007 there were approximately 679 deaths in Meherpur district due to violent attacks.

**Figure 3:**
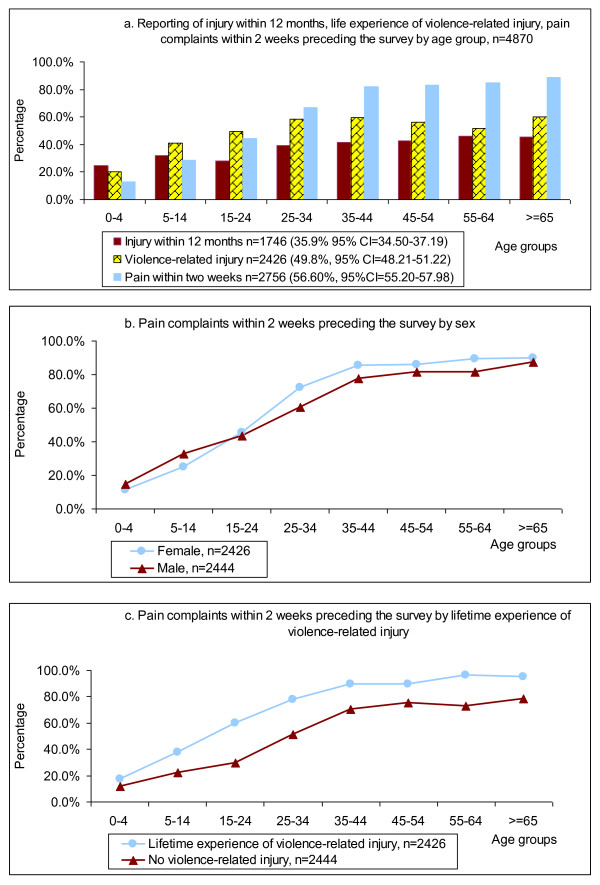
**Prevalence of injury, lifetime experience of violence-related injury and pain complaints by age groups**.

The reported pain complaints increased with age, reaching a plateau over the age of 35; there was little difference between men and women in the study population (Figure 3). More than 80% of the population over 35 years old reported experiencing pain within 2 weeks preceding the survey. With cluster effect (village) taken into account, the association between pain complaints and the lifetime experience of violence-related injury was positive for males in all age groups except for the 45-54 age group.

### Prevalence and geographical variation

Overall, 83.4% of households reported being exposed to at least two categories of OPV or human rights violations (Table 3): two-thirds of the households had experienced gunshots, shelling, or bombing in the neighbourhood; 75% of the households had witnessed a violent action against their friends or neighbours. Nearly 30% (n = 317) of families reported that at least one family member had been arrested or detained without warrant. Over 30% (n = 343) of families reported that at least one family member had been subjected to torture and other cruel, inhuman or degrading treatment or punishment and among them 182 (53.1%) were arrested or detained without warrant, 266 (77.4%) had been exposed to at least four categories of OPV and human rights violations, and 337 (98.3%) had been exposed to at least two categories. Thus, torture co-exists with other forms of OPV and human rights violations.

**Table 3:** Prevalence of organised crime and political violence (OPV) and human rights violations

Household experience	No. of households (%)
House search by legal authority or law enforcement agency	322 (29.3)
House occupied by legal authority or law enforcement agency	305 (27.8)
Forced evacuation or displacement	44 (4.0)
Gunshot, shelling, bombing in the neighbourhood	740 (67.3)
Illegal demolition of household property or food supply essential for survival	145 (13.2)
Victimised by liberation war with Pakistan	91 (8.3)

**Individual experience at household**	**No. of households (%)**

Saw relatives being arrested, assaulted, tortured, humiliated, injured or killed	707 (64.3)
Saw friend or neighbour being arrested, assaulted, tortured, humiliated, injured or killed	834 (75.9)
Arrest and detention without warrant or an order	317 (28.8)
Forced separation from family members	32 (2.9)
Kidnapping, trafficking, disappearance	35 (3.2)
Involvement in a combat or 'Cross-Fire' incidents	54 (4.9)
Extrajudicial execution by law enforcement agency	78 (7.1)
Forced labour by law enforcement agency	86 (7.6)
Experience of sexual harassment, molestation, rape or inserting blunt object into genital organ and/or rectum by members of law enforcement agency	9 (0.8)
Torture and other cruel, inhuman or degrading treatment or punishment	343 (31.2)

**Collective exposure to different categories of OPV and human rights violations**	**No. of households (%)**

0	51 (4.6)
1	132 (12.0)
2	212 (19.3)
2-4	338 (30.8)
4-6	214 (19.5)
6-8	90 (8.2)
8-10	50 (4.5)
10-12	12 (1.1)

Collective exposure reported by the sampled households, n = 1099

**Figure 4:**
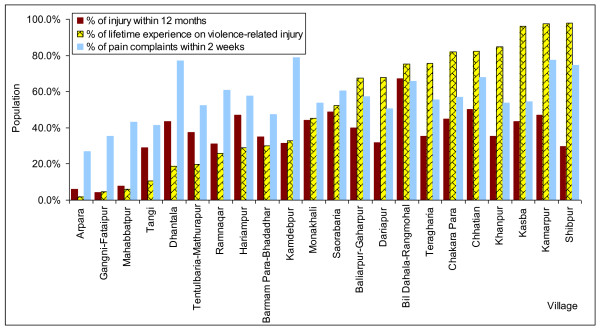
**Injury, lifetime experience of violence-related injury and pain complaints by village**. February--March 2008, n = 4870.

Variations in reporting torture and other cruel, inhuman or degrading treatment or punishment between different villages are presented in Figures 2 and 4. In Mahabbatpur, Gangni-Fataipur, and Tangi, no family reported a case of torture and other cruel, inhuman or degrading treatment or punishment, while over 30 families reported cases in Kasba, Kamarpur and Shipur, which are near the border with India. Variations in annual injury rate, prevalence of lifetime experience of violence-related injury, and pain complaints were also observed among the villages (Figure 4). Higher numbers of lifetime experience of violence-related injury were also reported from Meherpur Sadar upazila.

### Risk and protective factors

Meherpur Sadar upazila had a higher number of reported arrests and detentions (odds ratio [OR] = 1.58, 95% CI: 1.00-2.49, P = 0.05) and combat or cross-fire incidents (OR = 26.09, 95% CI: 27.5-247.28, P < 0.005). Meherpur Sadar and Mugibnagar upazila had significantly higher odds ratio of reported extrajudicial executions than Gangni upazila: Meherpur Sadar OR = 6.38, (95% CI: 1.01-40.40, P < 0.05); Mugibnagar OR = 5.63, (95% CI: 1.41-22.45, P < 0.05 (Table 4). There was fighting in Teragharua in Meherpur Sadar upazila, and 43 cases of extrajudicial execution were reported on this occasion.

**Table 4:** Exposure variables of organised crime and political violence (OPV) and human rights violations

Exposure	Variables	Arrest or detention				Torture, and other cruel, inhuman or degrading treatment or punishment				Participation in a combat and 'Cross-Fire'				Extrajudicial execution or murder by law enforcement agency			
		**No**	**Yes**	**OR (95% CI)**	**P value**	**No**	**Yes**	**OR (95% CI)**	**P value**	**No**	**Yes**	**OR (95% CI)**	**P value**	**No**	**Yes**	**OR (95% CI)**	**P value**
Involved in political party	Not involved	614	194	1	-	597	211	1	-	800	8	1	-	786	22	1	-
	Awami League	61	67	3.44 (2.25-5.26)	< 0.001	67	61	2.54 (1.15-5.61)	< 0.05	107	21	19.53 (4.71-80.91)	< 0.001	102	26	9.22(2.60-32.64)	< 0.001
	Bangladesh Nationalist Party (BNP)	81	46	1.85 (0.92-3.71)	0.083	65	62	2.92 (1.41-6.03)	< 0.005	103	24	21.62 (4.21-111.02)	< 0.001	100	27	9.15 (2.02-41.54)	< 0.005
	Jamaat-e-Islami Party	22	9	1.31 (0.68-2.50)	0.419	25	6	0.51 (0.15-1.75)	0.285	30	1	2.03 (0.29-14.04)	0.472	28	3	3.97 (1.05-15.04)	< 0.05
	Other	2	1	1.60 (0.16-15.64)	0.686	1	2	3.47 (0.31-38.33)	0.331	3	0	0	-	3	0	0	-

Social participation	Not involved	473	133	1	-	444	162	1	-	601	5	1	-	587	19	1	-
	Family member has participated in a demonstration, a strike, or a human rights rally	308	184	2.10 (1.57-2.81)	< 0.001	312	180	1.50 (0.88-2.57)	0.136	443	49	10.98 (5.07-23.78)	< 0.001	433	59	3.64 (1.25-10.64)	< 0.05

Inter-personal relationship	Not involved	240	55	1	-	214	81	1	-	289	6	1	-	286	9	1	-
	Family member has personal, financial or political conflict with other families	541	262	2.38 (1.80-3.14)	< 0.001	542	261	1.26 (0.71-2.21)	0.429	755	48	4.03 (2.23-7.27)	< 0.001	734	69	3.68 (1.99-6.78)	< 0.001
	No relationship	378	158	1	-	363	173	1	-	502	34	1	-	491	45	1	-
	Family member or friends work with a law enforcement agency	403	159	0.92 (0.67-1.27)	0.621	393	169	0.90 (0.65-1.24)	0.509	542	20	0.49 (0.29-0.81)	< 0.01	529	33	0.63 (0.31-1.29)	0.207

Upazila (sub-district)	Gangni upazila	355	94	1	-	329	120	1	-	447	2	1	-	441	8	1	-
	Mujibnagar upazila	113	87	2.62 (0.90-7.63)	0.078	105	95	2.38 (0.70-8.11)	0.165	197	3	2.23 (0.40-12.52)	0.362	181	19	5.63 (1.41-22.45)	< 0.05
	Meherpur upazila	314	136	1.58 (1.00-2.49)	0.05	322	128	1.08 (0.34-3.49)	0.894	401	49	26.09 (2.75-247.28)	< 0.005	399	51	6.38 (1.01-40.40)	< 0.05

Religion of the head of household	Muslim	754	304	1	-	729	329	1	-	981	77	1	-	981	77	1	-
	Hindu	18	7	0.80 (0.41-1.55)	0.511	16	9	1.01 (0.24-4.29)	0.992	24	1	0.73 (0.29-1.85)	0.507	24	1	0.53 (0.15-1.94)	0.339

Household income (Bangladesh taka: USD = 68:1)	0-2000 taka per month	193	56	1	-	178	71	1	-	246	3	1	-	239	10	1	-
	2001-4000 taka per month	377	150	1.41 (1.01-1.96)	< 0.05	375	152	1.12 (0.70-1.81)	0.632	504	23	4.50 (0.78-25.97)	< 0.05	489	38	2.00 (0.59-6.76)	0.263
	4001-8000 taka per month	159	82	1.84 (1.20-2.81)	< 0.005	150	91	1.56 (0.98-2.48)	0.059	218	23	10.35 (1.45-73.79)	< 0.05	214	27	3.35 (0.72-15.52)	0.122
	> 8000 taka per month	49	25	1.88 (1.01-3.48)	< 0.05	50	24	1.09 (0.62-1.94)	0.757	70	4	4.80 (1.54-14.96)	< 0.01	71	3	0.79 (0.16-3.97)	0.776

Generalised linear model adjusted to household income level, size of family and cluster effect of village

A multivariate model was built up and tested in a stepwise manner, taking parameters of univariate associations into account. Household serves as the unit of analysis and weighting of family size was adjusted. The risk for a family member to be arrested or detained was higher if the income level of the household was higher (Table 4). Adjusted to the household income level and random cluster effect of village, the likelihood for a family member to be arrested or detained was significantly higher if a member of this family: 1) was involved in party politics; 2) had ever participated in a demonstration, a strike or a human rights rally; 3) had a personal or financial conflict with other families (Table 4). A statistically significant increased risk was observed for families involved in the Awami League and for families whose members had ever participated in a demonstration, strike or human rights rally. Family members were more likely to be arrested or detained if the family had a conflict with other families.

The families whose members were involved in the Awami League or the BNP were also more likely to report experiences of torture and other cruel, inhuman or degrading treatment or punishment (Table 4). There was no correlation, however, between having conflicts with other families and the reporting of torture or other abuse experience.

Families whose members were involved in politics or had a conflict with other families in the community were also more likely to report that a family member experienced combat or cross-fire incidents or was extrajudicially executed by members of a law enforcement agency (Table 4). The odds ratio of reporting combat or cross-fire incidents was 19.53 for Awami League supporters and 21.62 for BNP supporters. The odds ratio of reporting extrajudicial execution of a family member was 9.22 for Awami League supporters, 9.15 for BNP supporters, and 3.97 for Jamaat-e-Islami Party supporters. An association was found between reporting combat or cross-fire incidents or extrajudicial execution, and participation in a political or social movement (a demonstration, a strike or a human rights rally). The reporting was also significantly higher among families that had conflicts with other families. There was a strong protective effect if relatives or friends of the family worked with a law enforcement agency: OR = 0.49, (95% CI: 0.29-0.81, P < 0.01).

### Consequences for family health

Annual injury rates, lifetime experiences of violence-related injury, and pain complaints within 2 weeks were statistically associated with an increase of collective exposure to OPV or human rights violations. A generalised linear model was adjusted for the cluster effects of village, location of dwelling, household income level, and whether the family member had ever participated in a political party, a demonstration, a strike or a human rights rally. Compared with families exposed to less than four categories of OPV or human rights violations, the family exposed to at least four categories of OPV or human rights violations yielded higher regression coefficients for the proportion of household members reporting injury within 12 months: 14.10, (95% CI: 4.23-23.97, P < 0.005). The regression coefficients were also higher for the proportion of household members reporting lifetime experience of violence-related injury: 22.36, (95% CI: 12.48-32.24, P < 0.001) and pain complaints within 2 weeks: 6.65, (95% CI: 0.51-12.80, P < 0.05).

### Financial burden of injury

Among 1,101 households, 522 families (47.5%) reported declining household income levels, 404 (36.7%) had borrowed money because of a major injury incident with a family member, and 181 of those 404 families were still in debt at the time of the survey. Loan amounts ranged from 100 to 150,000 Bangladeshi taka (1.5-2,205 USD). The average loan amount was 9,791 Bangladeshi taka (144 USD), which amounted to approximately two to three times the monthly household income of most families (70.4% of families had a monthly income of less than 4,000 Bangladeshi taka, that is, 2 USD per day). There was a heavy social and financial burden of having a high injury rate, which was related to collective exposure to OPV and human rights violations: 416 families reported that a family member had stopped working at least once in order to take care of an injured person (Table 5). Adjusted for income level of household, education and occupation of the head of household, village, and number of family members who had a lifetime experience of violence-related injury, the results showed that families exposed to at least two categories of OPV and human rights violations were likely to have declining household income levels due to the presence of an injured person: OR = 2.29, 95% CI: 1.22-4.31, P < 0.01. It was also more likely that the family had borrowed money (OR = 2.10, 95% CI: 1.24-4.25, P < 0.05) or that family members had stopped working in order to take care of the injured person (OR = 2.89, 95% CI: 1.37-6.11, P < 0.005).

**Table 5:** Financial and social burden due to an injury event

Financial and social burden	Household income declines due to the injury of family member				Family member stops working in order to take care of injured person				Family member stops schooling in order to take care of injured person or make up the loss of income				Family borrows money to pay for medication or to make up for the loss of income			
**Items**	**No**	**Yes**	**OR (95% CI)**	**P value**	**No**	**Yes**	**OR (95% CI)**	**P value**	**No**	**Yes**	**OR (95% CI)**	**P value**	**No**	**Yes**	**OR (95% CI)**	**P value**
No OPV and human rights violations	35	16	1	-	42	9	1	-	49	2	1	-	40	11	1	-
1 category of OPV and human rights violations	91	41	0.88 (0.43-1.82)	0.740	102	30	1.16 (0.50-2.71)	0.729	128	4	0.80 (0.14-4.58)	0.803	99	33	1.06 (0.48-2.37)	0.884
≥ 2 categories of OPV and human rights violations	450	465	2.29 (1.22-4.31)	< 0.01	538	377	2.89 (1.37-6.11)	< 0.005	842	73	1.64 (0.38-7.07)	0.509	555	360	2.10 (1.04-4.25)	< 0.05

Total	576	522			682	416			1,019	79			694	404		

Generalised linear model adjusted to education level, occupation of head of household, household income level, upazila and number of family members who reported lifetime experience of violence-related injury, n = 10

## Discussion

Case reports of OPV and human rights violations have been focused on individual experiences. Often, these are high profile cases and political bias and stance may influence case reporting, particularly in a society with high political tensions [19]. The problem of under-reporting is substantial. In addition, case reports do not consider the health impact of OPV (including psychological torture) on family members or the long-term economic and social consequences for the community. Epidemiological tools offer an opportunity to assess systematically all forms of OPV and human rights violations at the population level and estimate their short- and long-term effects on physical, psychological and social well-being of the individual, family and the community [20-22].

With a pervasive feeling of pain and the perceived or real threat of OPV, there are psychological consequences of violence-related trauma and trauma-related disorders for family members of victims [23-27]. Therefore, we used the household as a unit to study the prevalence, risk factors and impact of collective violence on the family. This is the first epidemiological survey to confirm the elevated prevalence of injury, lifetime experience of OPV-related injury and pain complaints, CMR and U5MR in relation to the level of exposure to OPV and human rights violations in a complex setting. Both CMR and U5MR in Meherpur district are higher than the UNICEF figures for Bangladesh in 2006 (Table 2). The CMR in Meherpur district (17.9 per 1,000) remained close to the rate observed during the liberation war in 1971 (18.9 per 1,000) and the rate was also the same as the CMR in Zimbabwe, ranking 12^th ^highest in the world in 2006. The CMR (0.49 per 10,000 per day) was also similar to that of most African countries at civil war (0.5 per 10,000 per day), while the U5MR (2.1 per 10,000 per day) had reached the WHO emergency threshold (2.0 per 10,000 per day). The violence-related mortality rate in Meherpur district was similar to the rate in a conflict zone in eastern Burma (Table 2)[28]. In addition, a majority of the study population suffered from pain. High mortality rate, injury rate and pain complaints of a population in the violence-prone areas should be taken as a warning sign of deteriorating public health.

Both qualitative and quantitative methods were used to determine the major risk factors for becoming a victim of OPV and human rights violations among the study population. Victimisation in Meherpur district is usually quite selective. A combination of individual, relational, community and societal factors, including variables such as age, household income level, area of residence, political party affiliation and patterns of social participation affect the risk of victimisation. Understanding these multilevel factors can help identify various opportunities for prevention [29].

Violence has remained inseparable from Bangladeshi politics and society. Bangladesh has gone through numerous political crises including two presidential assassinations, four coups, and at least 18 failed coup attempts since 1971 independence. The ongoing conflict between the leaders of the Awami League and the BNP is a prolongation of the three-decade hostility between these two families. The party elites instigated hatred of rival groups for political and economic gain; the struggle for power and resources between the two major parties has been blamed for the political polarisation [5,30]. In the last decade, multiparty democracy has been restored in Bangladesh. Although State Fragility Index and Polity scores have improved [31], the democracy is far from consolidated. A 5-level Political Terror Scale [32] and the Cingranelli-Richards Human Rights Dataset [33] coded the practice of human rights of 190 countries in a particular year based on the yearly reports of the "US State Department Report" and Amnesty International. Both showed that violations of civil and political rights in Bangladesh have been systematically increasing from the 1990s and have become a routine practice of the security forces since 2001. This has jeopardised the multiparty democracy in Bangladesh. State-organised violence is by nature instrumental in achieving specific goals; the nature of state-organised violence must always be considered while studying violence-related injury and trauma in complex settings and post-conflict settings. In the absence of an effective international monitoring mechanism and preventive measures in complex settings and post-conflict settings, OPV and human rights violations are likely to be widespread. A similar situation has been also reported for Kashmir, India [34-36] and Karachi, Pakistan [27,37-39]

The largest Islamic political party Jamaat-e-Islami Party was founded in pre-partition India in 1941. It has been against the independence of Bangladesh from Pakistan. One of the leaders of the Jamaat-e-Islami Party organised a militia in the Khulna division during the Bangladesh Liberation War in 1971 to assist the army of Pakistan. Until today, Khulna division, including Meherpur district, is still under the threat of Islamic extremists linked to Pakistan. The Jamaat-e-Islami Party has been in an alliance with the BNP and participated in a four-party coalition government during 2001-2006. Political polarisation is often blamed for attacks against Hindus because the main opposition party in 2001-2008, Awami League, has traditionally been the principal beneficiary of Hindu votes [2]. Our study produced no evidence, however, that followers of Hinduism among the study population were at increased risk of OPV and human rights violations. A study also noted that no case of violence on religious differences was reported in Karachi, Pakistan [27].

The prevalence of conflict between families in villages is extremely high in Meherpur district (Table 1). It is also known that, in Bangladesh, vengeance, family or land disputes, dowry disagreements and political conflicts are the major causes of clashes between families and villages [6,8,30]. Based on the narratives of victim families obtained from focus group discussions, families can bribe the police or army to attack members of rival families. People from families with the highest income levels have a relatively higher risk of being arrested or detained than people from the poorest families, but there is no evidence that they are more likely to be tortured or executed. No proof can be derived from our study; however, our findings support the public opinion collected from focus group discussions and recent ethnographic research that Bangladeshi police extort money from the population [5,26,40]. According to Transparency International, 71% of the accused paid bribes to the police at an average rate of 5,718 Bangladesh taka (84 USD). Bangladeshi police arrest people without warrants and then use torture to extract bribes from the victims [30,40]. The police offer to drop the charges in exchange for a bribe. If the negotiations do not yield anything lucrative, they torture the detainee. Richer families are more likely to arrange bribes to save victims from detention, and this could explain why the risk of being tortured or executed is lower for victims that come from the richest families [41].

Considerable geographical variation in OPV exposure was shown in the study areas, indicating 'hot spots' of violence and suggesting the responsibility of the local police force. The number of reported extrajudicial executions was higher in Meherpur Sadar upazila, and this tendency needs to be noticed. Traffic and trade between Bangladesh and India once flowed freely. Now, crossing an international border is highly regulated by the border security force. Criminals and terrorists also travel across the border, smuggling women, children or goods. The Bangladeshi government's decision to rely on the security force to tackle organised violence was initially widely welcomed; however, it soon came to be perceived as a convenient mechanism for unlawful income generation and political witch-hunting [5].

The leaders rely on the members of law enforcement agencies to wipe out the challengers to their power, and to crush those who have different political views and ideology or the criminals. The members of law enforcement agencies, however, have their own goals, independent from the leaders, and they could easily exploit the information and power advantage for their private interests. Pervasive corruption in the public sector in Bangladesh allows the members of law enforcement agencies to conceal their unlawful actions. The correlation of the level of human rights violations and sexual violence with the level of democracy and corruption has been examined and a similar pattern has been observed in India [42]. OPV in Meherpur district is incited by a mixture of various motivations: political goals of leaders and private interests of the perpetrators (that is, reward or enjoyment of the agent), which support the theory of a principal-agent problem [42-44]. In summary, when the politics of organised crime and the organised crime of politics are mingled in Bangladesh, the victims are both economically and politically deprived.

In another paper, we will provide comprehensive information on the results of subsequent OPV screening at the mobile clinic, which allowed us to better understand the time, place, type and amount of exposure and to assess the association between exposure to OPV or human rights violations and physical and emotional fitness and social functioning of an oppressed population.

A community-based rehabilitation project was implemented by BRCT in selected villages in nine districts of Khulna division from 2005 to 2008. It had a specific focus on human rights education, community health and community development. We have established a population baseline in Meherpur district, reshaped the model of above community-based intervention and developed implementation strategies based on the knowledge generated from this study.

### Limitations and strengths

A concern has been raised that carrying out such a survey can put the oppressed population at risk of state-organised violence. Therefore, it was absolutely crucial to work with a local NGO that had the capacity to provide both legal and health support to the victims. The BRCT and Task Force against Torture have provided legal support to victims, including making bail arrangements, providing legal advice and lobbying for legislative changes for many years. Both of them will be following up on the legal and health needs of victims once our study is completed. During the household survey, the majority of respondents were female. One of the strengths of our study is that the interviewers from the local NGO Manab Unnayan Kendra are very experienced in interviewing and providing consultation to victims of domestic violence and rape. Their experience helped to increase the survey response rate (99.9%). The household members were asked to remain to confirm the information provided. On the other hand, it made it embarrassing for the victims of sexual violence to reveal such an attack in front of their family members. Previous experience showed us that the victims of forced sexual contact or sexual torture were often in denial of tragedy or not ready to speak about it openly at the first or second approach by the social workers. They were likely to reach out for help afterwards. The brochures of the BRCT and Manab Unnayan Kendra were provided for their reference as resources that offer assistance or legal consultation for such types of victims. We believed that the numbers of victims of sexual violence in our survey were under-reported because of social stigma. The major limitations of this study are the cross-sectional and retrospective nature of the survey and there is concern about the accuracy and reliability of the survey participants' responses. Memory bias exists although a 10-year-recall is considered reliable [45,46]. There is selection bias in that only one person from each household was interviewed. The head or the spouse of the head of the household may not necessarily know everything that happened to family members. To reduce the bias in responding, the other available household members were asked to remain to verify information. It is also possible that people in the village misunderstood the definition of each category of OPV and human rights violations. Finally, many of these limitations stem from the difficulty of conducting research in a society that is polarised politically. Some people are afraid to talk about the political violence and some party supporters accuse each other. The victim families were self-reported, and they may have expected to receive humanitarian aid. To avoid such expectations, interviewers did not mention the vouchers and subsequent mobile clinic activity and explained that there would be no compensation for participation in the survey. The reporting of injury and pain would have likely increased if survey participants were expecting free medical treatment from the BRCT, which established a pain treatment centre in the neighbouring district in Khulna division.

## Conclusion

The baseline was established for collective exposure to OPV and human rights violations in the general population of Meherpur district. We identified a considerable number of silent victim families in the study area. The prevalence of household experience of different OPV and human rights violations was very high. The affected population suffered from violence-related injuries and traumas, which could be one of the root causes of poverty in rural areas in Bangladesh. The disaggregated geographical information enabled us to identify areas with different levels of exposure to OPV and human rights violations and to prioritise health and legal interventions. Both qualitative and quantitative methods should be employed to study collective violence in the complex setting in order to estimate the emergency level and complexity. This would help in developing an early warning network [29,47-49]. We hope the results of our study will assist in the advocacy effort, and we call for immediate multidisciplinary intervention and peace-building at the western border of Bangladesh.

## List of abbreviations

BNP: Bangladesh Nationalist Party; BRCT: Bangladesh Rehabilitation Centre for Trauma Victims; CMR: crude mortality rate; ICD: International Statistical Classification of Diseases and Related Health Problems; NGO: non-governmental organisation; OPV: organised crime and political violence; OR: odds ratio; Rehabilitation and Research Centre for Torture Victims; UNICEF: United Nations Children's Fund; USD: United States dollars; U5MR: under 5 mortality rate; WHO: World Health Organization.

## Competing interests

The authors declare that they have no competing interests.

## Authors' contributions

SJW participated in the design of the study, conducted the field work, analysed and interpreted the data and drafted the manuscript. EM and JM participated in the conception of the work, helped to draft the manuscript and provided critical review at all stages. All authors have read and approved the final version of the manuscript.

## Pre-publication history

The pre-publication history for this paper can be accessed here:

http://www.biomedcentral.com/1472-698X/9/29/prepub
